# Outage Probability Performance Prediction for Mobile Cooperative Communication Networks Based on Artificial Neural Network

**DOI:** 10.3390/s19214789

**Published:** 2019-11-04

**Authors:** Han Wang, Lingwei Xu, Xianpeng Wang

**Affiliations:** 1 College of Physical Science & Engineering, Yichun University, Yichun 336000, China; 2 Institute of Data Science, City University of Macau, Macau 999078, China; 3 Department of Information Science & Technology, Qingdao University of Science & Technology, Qingdao 266061, China; 4 Key Laboratory of Opto-Technology and Intelligent Control, Ministry of Education, Lanzhou Jiaotong University, Lanzhou 730070, China; 5 State Key Laboratory of Marine Resource Utilization in South China Sea, Hainan University, Hakou 570228, China; wxpeng2016@hainu.edu.cn

**Keywords:** mobile cooperative communication, outage probability, performance prediction, BP neural network

## Abstract

This paper investigates outage probability (OP) performance predictions using transmit antenna selection (TAS) and derives exact closed-form OP expressions for a TAS scheme. It uses Monte-Carlo simulations to evaluate OP performance and verify the analysis. A back-propagation (BP) neural network-based OP performance prediction algorithm is proposed and compared with extreme learning machine (ELM), locally weighted linear regression (LWLR), support vector machine (SVM), and BP neural network methods. The proposed method was found to have higher OP performance prediction results than the other prediction methods.

## 1. Introduction

Mobile applications have grown explosively in recent years, leading to an increased interest in mobile communication systems [1,2,3]. Relay-assisted mobile cooperative communication is an inevitable trend for future mobile networks, especially with regard to reliability [4,5,6]. In a study by the authors of [4], a multi-antenna decode-and-forward relay was used to assist a two-user non-orthogonal multiple access system and ensure secure transmission. For trusted and untrusted scenarios, secure relaying was considered in non-orthogonal multiple access [5]. A beamforming design was investigated for cooperative secure transmission in cognitive two-way relay networks [6].

As a promising technology, multiple-input-multiple-output (MIMO) can improve the performance of mobile cooperative communication. Massive MIMO was combined with general orthogonal precoding for high mobility scenarios in [7]. A minimum mean square error (MMSE) detector was used for channel estimation of massive MIMO systems [8]. In [9], the pairwise error probability (PEP) of the massive multiuser MMSE-MIMO systems was derived. However, the hardware complexity of MIMO also increased. Transmit antenna selection (TAS) is a practical option to reduce hardware complexity. It has also been attracting the attention of academicians [10,11,12]. Based on the asymptotic theory on order statistics, the authors of Ref. [13] derived the asymptotic upper capacity bounds of massive MIMO system with TAS over Rayleigh fading channels. A TAS strategy was investigated for full-duplex distributed antenna systems in [14]. Based on Monte Carlo Tree Search (MCTS), a self-supervised learning method was proposed to solve the antenna selection problem for a massive MIMO system [15]. TAS and extreme value theory were used to investigate the asymptotic behavior of spectrum-sharing systems in [16].

To data, cooperative communication and TAS technologies only consider Rayleigh and Nakagami fading channels. In reality, due to the complex and variable environments, the fading channels for mobile cooperative communication are more complicated than Rayleigh and Nakagami fading channels [17,18]. The *N*-Nakagami model is adopted in mobile cooperative communication [19,20,21]. The outage probability (OP) performance is essential for the design and evaluation of mobile cooperative communication networks over *N*-Nakagami channels. Therefore, predicting OP performance on time and then providing corresponding evaluation can effectively guarantee communication quality. However, performance prediction is the foremost task in the evaluation of the mobile cooperative communication networks. There is a lack of research on OP performance prediction of mobile cooperative communication networks with TAS.

Motivated by the above discussion, we investigate OP performance prediction with variable-gain amplify-and-forward (AF) relaying and TAS. The main contributions of this paper are as follows:

1. For the TAS scheme, we derive the novel exact closed-form mathematical OP expressions.
2. Based on the derived OP expressions, a back-propagation (BP) neural network-based OP performance prediction algorithm is proposed. We use the OP theoretical results to generate training data. We test extreme learning machine (ELM), locally weighted linear regression (LWLR), support vector machine (SVM), and BP neural network methods.
3. Through Monte-Carlo simulations, we verify the derived OP expressions. Compared with ELM, LWLR, and SVM methods, the Monte-Carlo results verify that our method can consistently achieve higher prediction results.

This paper is organized as follows: Section 2 provides the related work. The system model is presented in Section 3. The OP performance of the TAS scheme is investigated in Section 4. Based on the BP neural network, we propose an OP performance prediction algorithm in Section 5. OP performance is evaluated in Section 6. Section 7 offers concluding remarks.

## 2. Related Work

Cooperative communication has been proposed to improve the performance of mobile communication networks. The system performance of MIMO AF cooperative networks over the shadowed-Rician fading model was investigated in [22]. The OP performance of mobile cooperative networks was investigated in [23]. The Stackelberg game was exploited for a hybrid satellite-terrestrial cooperative network, and the harmonic mean function was used to select the best relay node in [24]. The error performance of the spatial modulation system over spatially correlated Rayleigh channels was presented in [25]. 

TAS is widely employed in cooperative networks to reduce hardware complexity. In [26], a novel TAS strategy was proposed for full-duplex AF relaying over the Rayleigh model. To reduce multiuser interference, the TAS mechanism was introduced into constant envelope pre-coding [27]. In [28], a TAS-maximal ratio combining relay system investigated the effect of channel estimation error. In [29], to reduce hardware complexity, two TAS schemes were proposed for secure transmission using precoding-aided spatial modulation.

Traditionally, performance evaluation is achieved by mathematical superposition, approximation, and fitting. These methods are faced with oversimplified real-world issues. Machine learning techniques have overcome these issues and are widely used in performance prediction [30]. In [31], a LWLR method was proposed to predict the damping ratio of a dominant mode online.SVM regression model was used to propose localizing algorithms for large-scale wireless sensor networks in [32]. A novel evolutionary algorithm was proposed for data classification problem with ELM in [33]. Because of good nonlinear prediction ability, a BP neural network model is more suitable for performance prediction. By using a BP neural network, Ref. [34] proposed a monitoring method of total seed mass. In [35], the BP network was used to obtain a direct readout of the applied force. To predict the telecommunication customer churn, Ref. [36] used a particle classification method to optimize the BP network. The authors of Ref. [37] employed BP network for a high accuracy channel estimation in secure cooperative transmission. In [38], the BP network was used to predict the Rayleigh fading channel.

## 3. System Model

**Figure 1:**
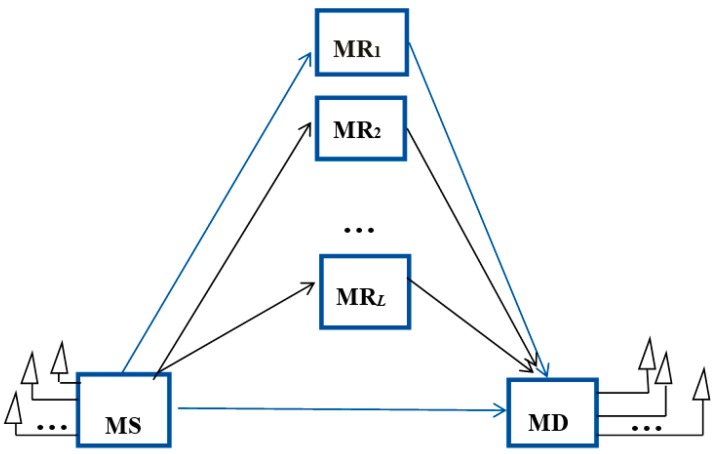
The system model.

Figure 1 presents the system model, which includes a mobile source (MS) node with *N*_t_ antennas, a mobile destination (MD) node with *N*_r_ antennas, and *L* mobile relay (MR) nodes. The nodes operate in half-duplex mode. The *L*MR nodes utilize their individual uplink channel state information (CSI) to select the best MR. The best MR node participates in collaboration. The MD node calculates the received signal-to-noise ratio (SNR) from the best MR node. The MD node orders the received SNR from *N*_t_ antennas, and then feedbacks the selected antenna to the MS node.

The channel coefficient *h* = *h_k_*, *k*∈{SD*ij*, SR*il*, RD*lj*}, *i*∈(1,*N*_t_), *j*∈(1,*N*_r_), *l*∈(1,*L*). The amplitude of *h* follows *N*-Nakagami distribution [19]. The total power is *E*. 

In the first timeslot, MS*_i_* transmits the signal *a*. MD*_j_* and MR*_l_* receive the signals *r*_SD*ij*_ and *r*_SR*il*_ as:

(1) $$r_{\mathrm{SD}ij}=\sqrt{KE}h_{\mathrm{SD}ij}a+n_{\mathrm{SD}ij}$$

(2) $$r_{\mathrm{SR}il}=\sqrt{G_{\mathrm{SR}il}KE}h_{\mathrm{SR}il}a+n_{\mathrm{SR}il}$$

where *G*_SD*ij*_ = 1 is the relative gain of MS→MD, *G*_SR*il*_ is the relative gain of MS*_i_*→MR*_l_*. *n*_SR*il*_ and *n*_SD*ij*_ have mean 0 and variance *N*_0_/2. *K* is the power allocation parameter, which controls power distribution between the MS and MR. For MS, the power is *KE.* For MR, the power is (1−*K*)*E*, *K*∈(0,1).

In the second timeslot, with AF method, MD*_j_* receives the signal as:

(3) $$r_{\mathrm{RD}lj}=\sqrt{c_{ilj}E}h_{\mathrm{SR}il}h_{\mathrm{RD}lj}a+n_{\mathrm{RD}lj}$$

where *G*_RD*lj*_ is the relative gain of MR*_l_*→MD*_j_*, *n*_RD*lj*_ has mean 0 and variance *N*_0_/2. *c_ilj_* is given as [39]:

(4) $$c_{ilj}=\frac{K(1-K)G_{\mathrm{SR}il}G_{\mathrm{RD}lj}E/N_{0}}{1+KG_{\mathrm{SR}il}{\left|h_{\mathrm{SR}il}\right|}^{2}E/N_{0}+(1-K)G_{\mathrm{RD}lj}{\left|h_{\mathrm{RD}lj}\right|}^{2}E/N_{0}}$$

where *N*_0_ is noise power.

MD*_j_* calculates the received signal-to-noise ratio (SNR) as:

(5) $$\gamma_{ij}=\max(\gamma_{\mathrm{SD}ij},\gamma_{\mathrm{SRD}ij})$$

(6) $$\gamma_{\mathrm{SD}ij}=\frac{K{\left|h_{\mathrm{SD}ij}\right|}^{2}E}{N_{0}}=K{\left|h_{\mathrm{SD}ij}\right|}^{2}\bar{\gamma }$$

(7) $$\gamma_{\mathrm{SRD}ij}=\underset{1\leq l\leq L}{\max}(\frac{\gamma_{\mathrm{SR}il}\gamma_{\mathrm{RD}lj}}{1+\gamma_{\mathrm{SR}il}+\gamma_{\mathrm{RD}lj}})$$

where *γ*_SRD*ij*_ is the SNR of MS→MR→MD link,*γ*_SD*ij*_ is the SNR of MS→MD link,*γ*_SR*il*_ is the SNR of MS*_i_*→MR*_l_* link, and*γ*_RD*lj*_ is the SNR of MR*_l_*→MD*_j_* link.

(8) $$\gamma_{\mathrm{SR}il}=\frac{G_{\mathrm{SR}il}K{\left|h_{\mathrm{SR}il}\right|}^{2}E}{N_{0}}=G_{\mathrm{SR}il}K{\left|h_{\mathrm{SR}il}\right|}^{2}\bar{\gamma }$$

(9) $$\gamma_{\mathrm{RD}lj}=\frac{(1-K)G_{\mathrm{RD}lj}{\left|h_{\mathrm{RD}lj}\right|}^{2}E}{N_{0}}=(1-K)G_{\mathrm{RD}lj}{\left|h_{\mathrm{RD}lj}\right|}^{2}\bar{\gamma }$$

It is difficult to obtain the closed-form solution to *γ*_SRD*ij*_. With the help of [40,41], we obtain an upper bound of *γ*_SRD*ij*_ as:

(10) $$\gamma_{\mathrm{SRD}ij}<\gamma_{\mathrm{up}ij}=\underset{1\leq l\leq L}{\max}(\min(\gamma_{\mathrm{SR}il},\gamma_{\mathrm{RD}lj}))$$

MD calculates the received SNR as:

(11) $$\gamma_{{\mathrm{SC}}_{i}}=\underset{1\leq j\leq N_{r}}{\max}(\gamma_{ij})$$

where $\gamma_{ij}=\max(\gamma_{\mathrm{SD}ij},\gamma_{\mathrm{up}ij})$.

We select *g* of TAS scheme as:

(12) $$g=\underset{1\leq i\leq N_{t}}{\max}(\gamma_{{\mathrm{SC}}_{i}})=\underset{1\leq i\leq N_{t},1\leq j\leq N_{r}}{\max}(\gamma_{ij})$$

## 4. The OP of Optimal TAS Scheme

We obtain the OP as:

(13) $$\begin{array}{l} F_{\mathrm{optimal}}=\mathrm{Pr}(\underset{1\leq i\leq N_{t},1\leq j\leq N_{r}}{\max}(\gamma_{ij})<\gamma_{\mathrm{th}})\\ ={\left(\mathrm{Pr}(\gamma_{\mathrm{SD}}<\gamma_{\mathrm{th}})\mathrm{Pr}(\gamma_{\mathrm{up}}<\gamma_{\mathrm{th}})\right)}^{N_{t}\times N_{r}}\\ ={\left(V_{1}V_{2}\right)}^{N_{t}\times N_{r}} \end{array}$$

where *γ*_th_ is a given threshold,*γ*_up_ is the upper bound of*γ*_SRD._

The *V*_1_ is evaluated as:

(14) $$\begin{array}{l} V_{1}=\mathrm{Pr}(\gamma_{\mathrm{SD}}<\gamma_{\mathrm{th}})\\ =\frac{1}{\prod_{d=1}^{N}\Gamma (m_{d})}G_{1,N+1}^{N,1}\left[\frac{\gamma_{\mathrm{th}}}{\bar{\gamma_{\mathrm{SD}}}}\prod_{d=1}^{N}\frac{m_{d}}{\Omega_{d}}|{}_{m_{1},\ldots ,m_{N},0}^{1}\right] \end{array}$$

(15) $$\bar{\gamma_{\mathrm{SD}}}=K\bar{\gamma }$$

where the *G*[·] is the Meijer G function, which is given as [19].

(16) $$\begin{array}{l} G_{p,q}^{m,n}\left[z|{}_{b_{1},\ldots ,b_{q}}^{a_{1,\ldots ,}a_{p}}\right]\\ =\frac{1}{j2\pi }\int_{\xi }\frac{\prod_{i=1}^{m}\Gamma (b_{i}+s)\prod_{i=1}^{n}\Gamma (1-a_{i}-s)}{\prod_{i=n+1}^{p}\Gamma (a_{i}+s)\prod_{i=m+1}^{q}\Gamma (1-b_{i}-s)}z^{-s}ds \end{array}$$

Next, *V*_2_ is evaluated as:

(17) $$\begin{array}{ll} V_{2} & =\mathrm{Pr}(\gamma_{\mathrm{up}}<\gamma_{\mathrm{th}})\\ & =\mathrm{Pr}(\underset{1\leq l\leq L}{\max}(\min(\gamma_{\mathrm{SR}},\gamma_{\mathrm{RD}}))<\gamma_{\mathrm{th}})\\ & =\mathrm{Pr}{\left(\min(\gamma_{\mathrm{SR}},\gamma_{\mathrm{RD}})<\gamma_{\mathrm{th}}\right)}^{L} \end{array}$$

where $\bar{\gamma_{\mathrm{SR}}}=KG_{\mathrm{SR}}\bar{\gamma }$, $\bar{\gamma_{\mathrm{RD}}}=(1-K)G_{\mathrm{RD}}\bar{\gamma }$, and

$$\begin{array}{l} \mathrm{Pr}(\min(\gamma_{\mathrm{SR}},\gamma_{\mathrm{RD}})<\gamma_{\mathrm{th}})\\ =1-\mathrm{Pr}(\min(\gamma_{\mathrm{SR}},\gamma_{\mathrm{RD}})>\gamma_{\mathrm{th}})\\ =1-\mathrm{Pr}(\gamma_{\mathrm{SR}}>\gamma_{\mathrm{th}},\gamma_{\mathrm{RD}}>\gamma_{\mathrm{th}})\\ =1-(1-\mathrm{Pr}(\gamma_{\mathrm{SR}}<\gamma_{\mathrm{th}}))(1-\mathrm{Pr}(\gamma_{\mathrm{RD}}<\gamma_{\mathrm{th}}))\\ =\mathrm{Pr}(\gamma_{\mathrm{SR}}<\gamma_{\mathrm{th}})+\mathrm{Pr}(\gamma_{\mathrm{RD}}<\gamma_{\mathrm{th}})-\mathrm{Pr}(\gamma_{\mathrm{SR}}<\gamma_{\mathrm{th}})\mathrm{Pr}(\gamma_{\mathrm{RD}}<\gamma_{\mathrm{th}})\\ =\frac{1}{\prod_{t=1}^{N}\Gamma (m_{t})}G_{1,N+1}^{N,1}\left[\frac{\gamma_{\mathrm{th}}}{\bar{\gamma_{\mathrm{SR}}}}\prod_{t=1}^{N}\frac{m_{t}}{\Omega_{t}}|{}_{m_{1},\ldots ,m_{N},0}^{1}\right]+\frac{1}{\prod_{tt=1}^{N}\Gamma (m_{tt})}G_{1,N+1}^{N,1}\left[\frac{\gamma_{\mathrm{th}}}{\bar{\gamma_{\mathrm{RD}}}}\prod_{tt=1}^{N}\frac{m_{tt}}{\Omega_{tt}}|{}_{m_{1},\ldots ,m_{N},0}^{1}\right]\\ -\frac{1}{\prod_{t=1}^{N}\Gamma (m_{t})\prod_{tt=1}^{N}\Gamma (m_{tt})}G_{1,N+1}^{N,1}\left[\frac{\gamma_{\mathrm{th}}}{\bar{\gamma_{\mathrm{SR}}}}\prod_{t=1}^{N}\frac{m_{t}}{\Omega_{t}}|{}_{m_{1},\ldots ,m_{N},0}^{1}\right]\times G_{1,N+1}^{N,1}\left[\frac{\gamma_{\mathrm{th}}}{\bar{\gamma_{\mathrm{RD}}}}\prod_{tt=1}^{N}\frac{m_{tt}}{\Omega_{tt}}|{}_{m_{1},\ldots ,m_{N},0}^{1}\right] \end{array}$$

## 5. Outage Probability (OP) Performance Prediction Based on BP Neural Network

### 5.1. Input and Output Selection

By the derived closed-form OP expressions, we can see that OP performance is affected significantly by *m*, *N*, *G* and *K*. We use *m*, *N*, *G,* *K* and other parameters as indicators. The input *X* includes 17 indicators, the output *y* is the corresponding OP performance obtained by Equation (13). The 17 indicators are*m*_SR1_, *m*_RD1_, *m*_SD1_, *m*_SR2_, *m*_RD2_, *m*_SD2_, *G*_SR_, *G*_RD_, *N*_SR1_, *N*_RD1_, *N*_SD1_, *N*_SR2_, *N*_RD2_, *N*_SD2_, *K*, *γ*_th_, $\bar{\gamma }$. Datasets are given as {*T_i_*}, *i* = 1,2,...,*P*. *T_i_* = (*X_i_*, *y_i_*). *X_i_* is given as:

(18) $$X_{i}=\left(x_{i1},x_{i2},\ldots ,x_{i17}\right)$$

### 5.2. BP Neural Network Structure

**Figure 2:**
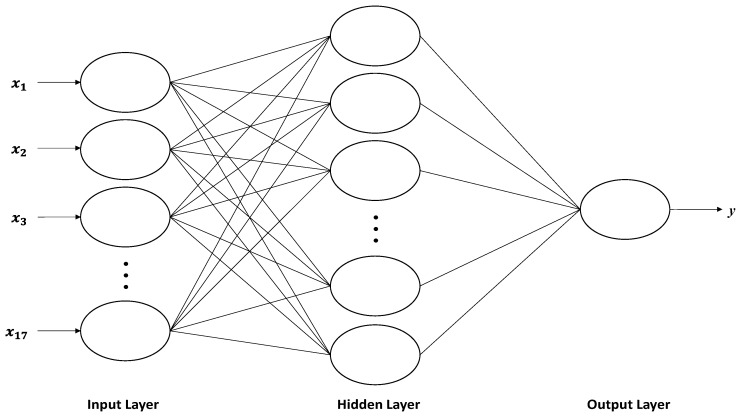
The back-propagation (BP) neural network structure.

The BP neural network is a kind of multi-layer pre-feedback artificial neural network. It changes its internal states according to the inputs, and produce outputs depending on the inputs and activation function. Figure 2 shows the BP neural network. It has three layers, namely the input layer, the hidden layer, and the output layer. For the input layer, there are 17 neurons. For the hidden layer, there are *q* neurons. For the output layer, there is 1 neuron. The network is formed by connecting the neurons in different layers, resulting in a directed and weighted graph. For the input and hidden layers, *w_ij_* is the weight coefficient, *b_j_* is the bias value. For the hidden and output layers, *v_j_* is the weight coefficient, *θ* is the bias value.

For the hidden layer, input is given as:

(19) $$s_{j}=\sum_{i=1}^{17}w_{ij}x_{i}+b_{j},j=1,2,\ldots ,q$$

The output is given as:

(20) $$c_{j}=f\left(s_{j}\right)$$

where *f*(x) is the activation function.

For the output layer, input is given as:

(21) $$\beta =\sum_{j=1}^{q}v_{j}c_{j}+\theta$$

The output is given as:

(22) y=f(β)

The output error *EE* is given as:

(23) $$EE=\sum_{h=1}^{P}{\left(d_{}^{h}-y_{}^{h}\right)}^{2}$$

where *y^h^* is the output for *h-*th data, and *d^h^* is the desired output.

### 5.3. The Flowchart of OP Performance Prediction Algorithm

**Figure 3:**
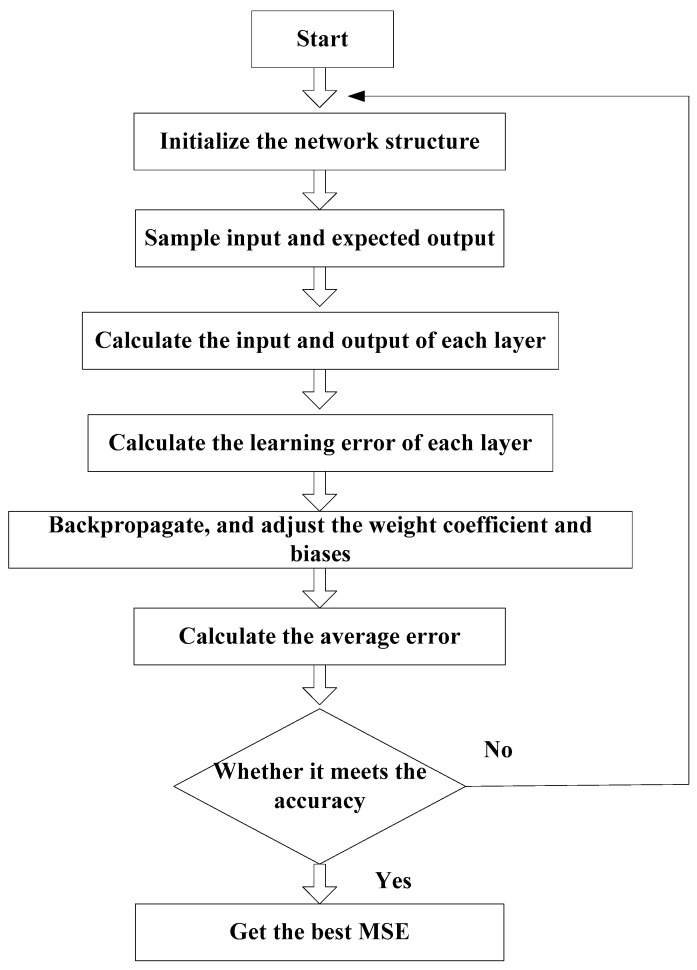
The flowchart of the outage probability (OP) performance prediction algorithm.

Figure 3 shows the flowchart of the OP performance prediction algorithm.

### 5.4. Metric

We use mean squared error (MSE) to evaluate the performance of different methods. MSE is computed as follows:

(24) $$\mathrm{MSE}=\frac{\sum_{h=1}^{PP}{\left(d_{}^{h}-y_{}^{h}\right)}^{2}}{PP}$$

where *PP* is the number of testing data.

## 6. Numerical Results

In this section, *E* = 1.*μ* = *G*_SR_/*G*_RD_.

**Figure 4:**
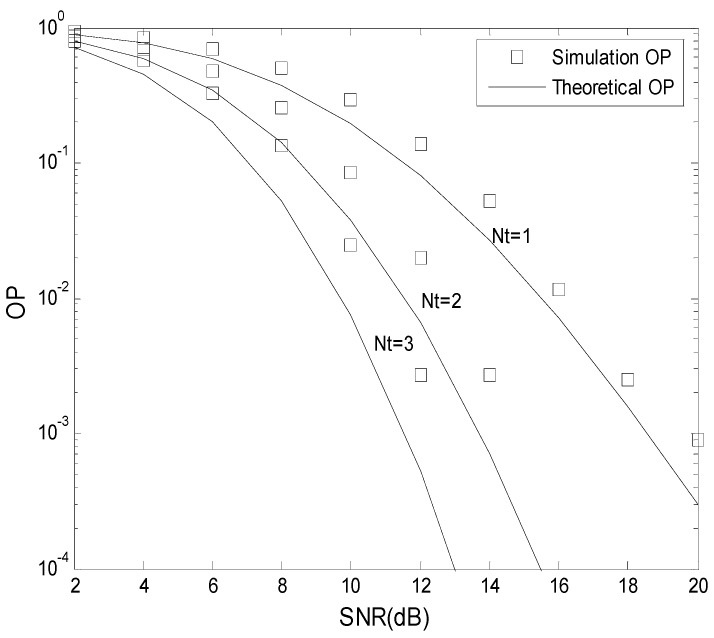
The OP performance of the transmit antenna selection (TAS) scheme.

Figure 4 presents the OP performance of the TAS scheme. Table 1 gives the parameters employed. From Figure 4, we see that the Monte-Carlo results and analytical results are similar. The OP is improved as *N*_t_ increased.

**Figure 5:**
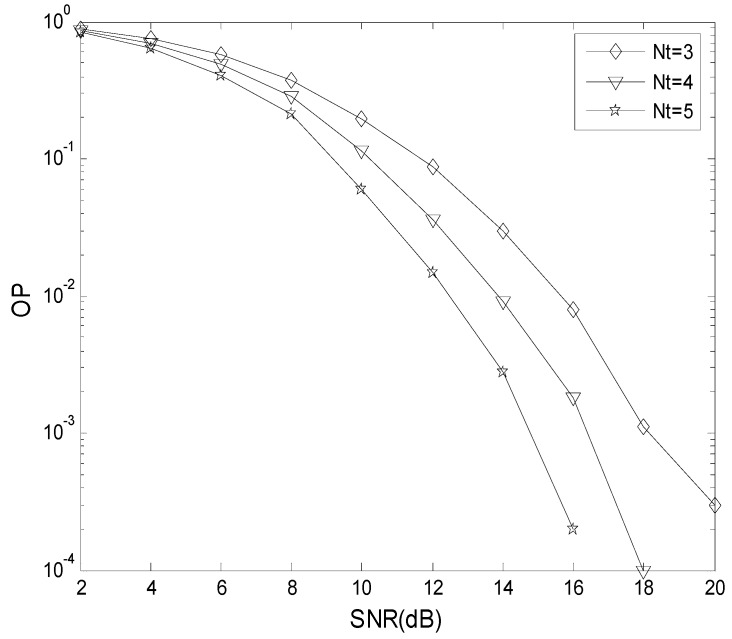
The effect of *N*_t_ on OP performance.

Figure 5 presents the effect of *N*_t_ on the OP performance. Table 2 gives the parameters employed. From Figure 5, with *N*_t_ increased, the OP decreases. When SNR=12dB, the OP is 8.6 × 10^−2^ with *N*_t_ = 3, 3.6 × 10^−2^ with *N*_t_ = 4, and 1.5 × 10^−2^ with *N*_t_ = 5.

**Figure 6:**
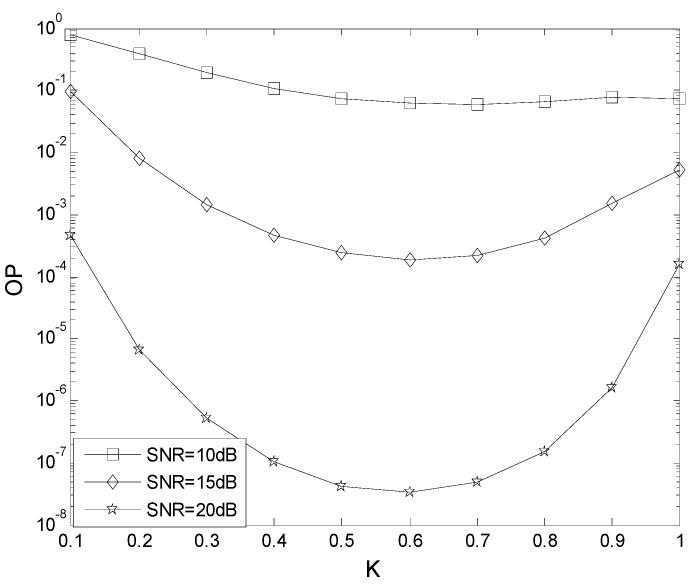
The effect of *K* on OP performance.

Figure 6 presents the effect of *K* on OP performance. Table 3 gives the parameters employed. From Figure 6, with SNR increased, the OP decreases. When *K* = 0.5, the OP is 3.8 × 10^−2^ with SNR = 10 dB, 2.0 × 10^−4^ with SNR = 15 dB, and 9.0 × 10^−8^ with SNR = 20 dB.

**Figure 7:**
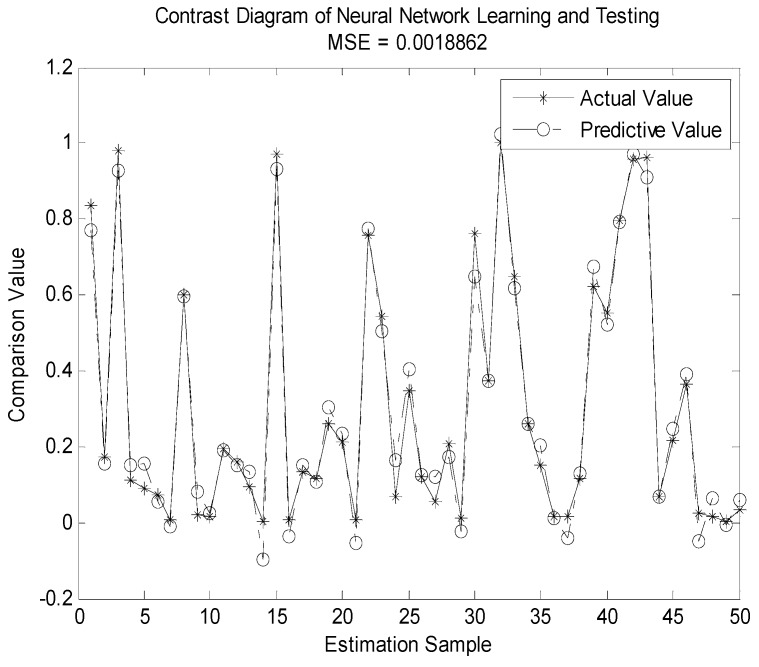
Actual and predictive outputs of the BP neural network.

**Figure 8:**
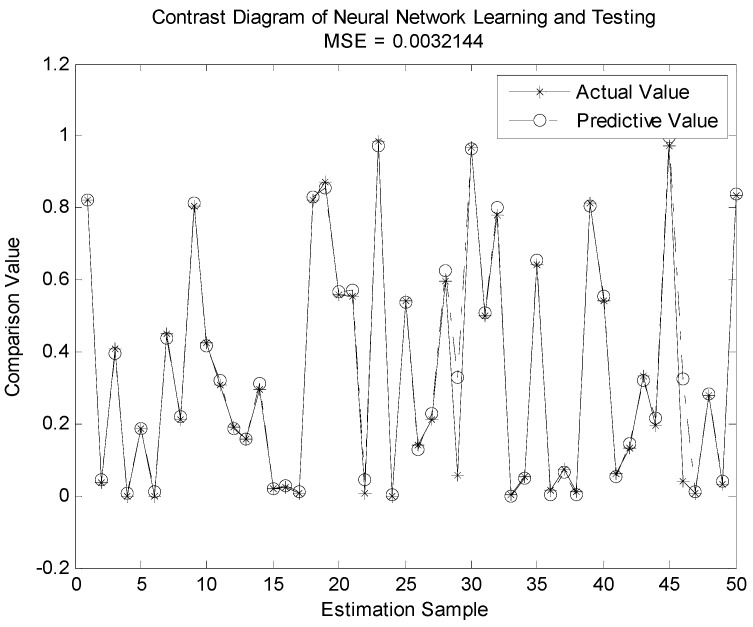
Actual and predictive outputs of extreme learning machine (ELM).

**Figure 9:**
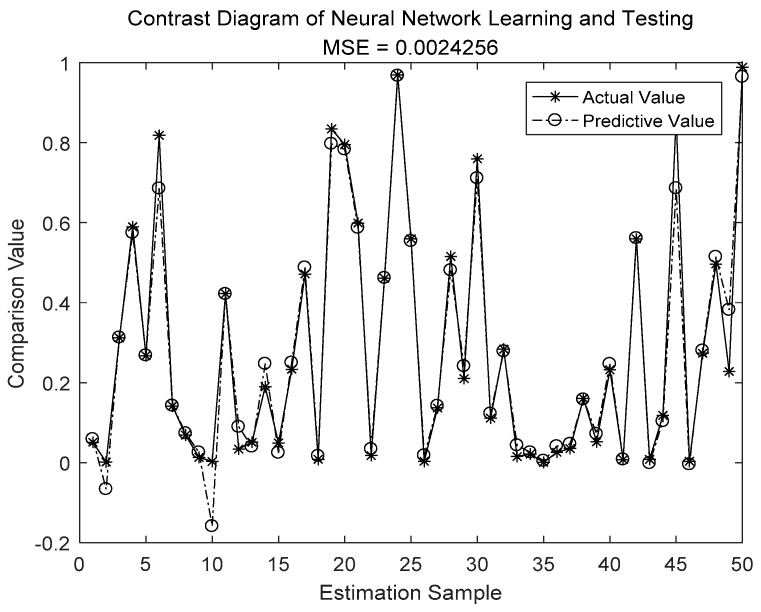
Actual and predictive outputs of support vector machine SVM.

**Figure 10:**
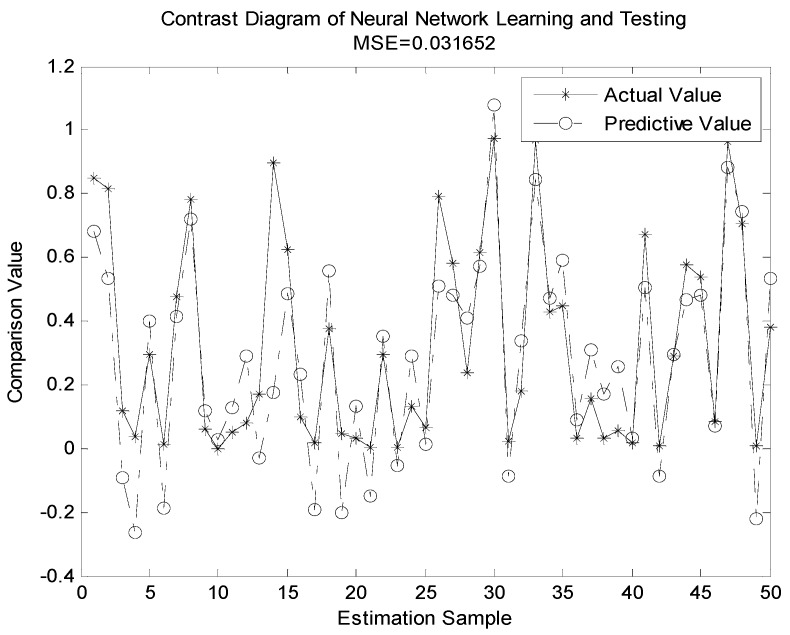
Actual and predictive outputs of locally weighted linear regression (LWLR).

In Figure 7, Figure 8, Figure 9 and Figure 10, we compare the BP neural network with LWLR [42], SVM [43], and ELM [44] methods.

(1) The LWLR [42] model is as follows:
  (25) $$\min\;\;\;\;\;\;\;\;\;\;\;\;f(\theta )=\sum_{k}\phi^{\left(k\right)}{\left(y^{\left(k\right)}-\theta^{T}X^{\left(k\right)}\right)}^{2}$$
  (26) $$\theta =(\theta_{0},\theta_{1},\cdots ,\theta_{n})$$
  (27) $$\phi^{\left(k\right)}=\exp\left(-\frac{{\left(X^{\left(k\right)}-X\right)}^{T}(X^{\left(k\right)}-X)}{2\tau^{2}}\right)$$
  where *θ* denotes the coefficient vector of the linear equation, *τ* is the bandwidth parameter.
(2) The SVM [43] model is as follows
  (28) $$\begin{array}{l} \min\;\;\;\;\;\;\;\;\;\;\;\;\frac{1}{2}{\left\|w\right\|}^{2}+c\sum_{i=1}^{n}\varepsilon_{i}\\ \begin{array}{cc} s.t. & y_{i}\sum_{i=1}^{m}KK(X_{i}^{T},X_{i}^{})\geq 1-\varepsilon_{i} \end{array} \end{array}$$
  where *w* is the adjustable weight,${\left\|w\right\|}^{2}$ is the Euclidean norm of the vector, *ε_i_* is the slack variable, and *c* is the penalty parameter. *KK*() is the kernel function, which has an important parameter g.
(3) ELM [44]: ELM has the same framework as the BP neural network. The input weight of ELM is subject to random assignment by a certain distribution function, and the output weight is directly calculated via the least squares method. The hidden layer has q neurons. Compared with the BP neural network, the training and recognition processes of ELM are rapid.

The parameters for the four different methods are given in Table 4. The number of train sets is 950, the number of test sets is 50. From Figure 7, Figure 8, Figure 9 and Figure 10, we see that the MSE of the BP neural network is 0.0018862, which is lower than that of the LWLR, SVM, and ELM methods. Compared to the LWLR, SVM, and ELM methods, our method can consistently achieve higher OP performance prediction results.

Table 5 shows the running time and MSE comparison for the four methods. In Table 5, we see that, compared to ELM, BP has a longer running time, but its performance is better than ELM. In addition, compared to SVM and LWLR, BP has a shorter running time and a smaller MSE. This is because the LWLR is not suitable for complex nonlinear data, the SVM has difficulty solving multi-class prediction problems, and the weights of ELM are generated randomly and maintained through the whole training process. The BP algorithm has strong nonlinear analytical abilities and robustness for multi-class prediction problems. A comprehensive comparison shows that BP is the best.

**Figure 11:**
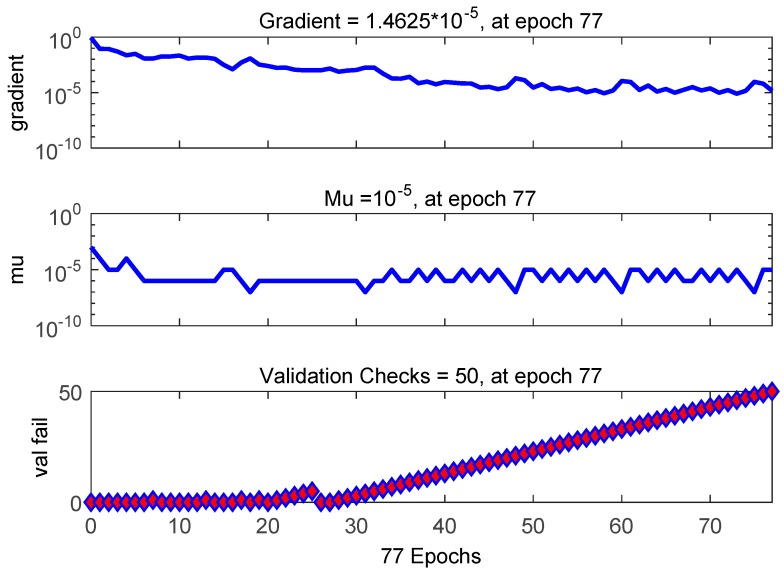
Training state of the BP neural network.

In Figure 11, we obtain the training state and see how the gradient changes with increase in the number of iterations.

**Figure 12:**
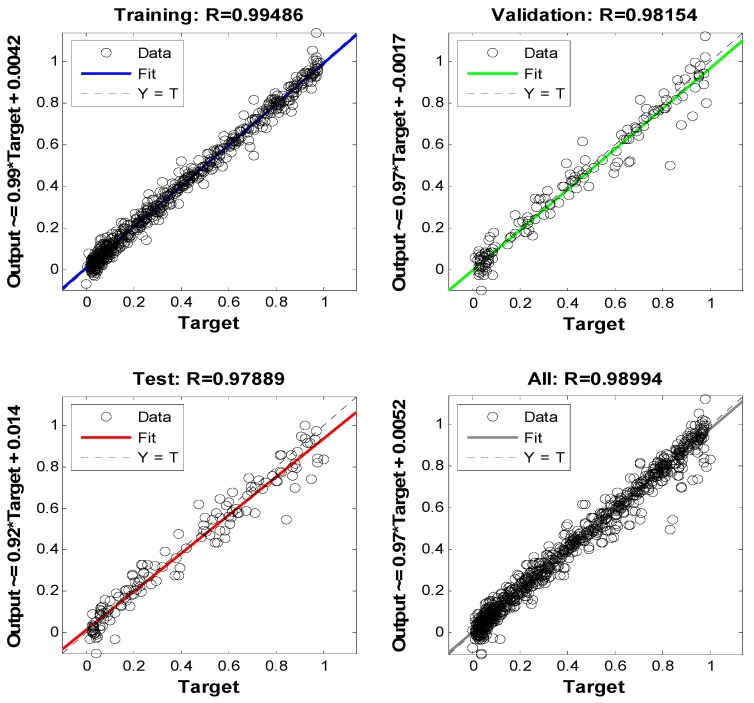
Regression of the BP neural network.

The regression results are shown in Figure 12. In each plot, the relationship between the targets and outputs is indicated by correlation coefficient *R*. In Figure 12, *R* is 0.98994, which indicates that our method has a good prediction capability.

## 7. Conclusions

In this paper, we derived exact closed-form OP expressions for AF relaying. To verify our proposed analysis, the theoretical results obtained were compared with Monte-Carlo simulation results. The effect of *K* and *N*_t_ on OP performance was also investigated. To predict OP performance, a BP neural network-based OP performance prediction algorithm was proposed. When compared with the LWLR, SVM, and ELM methods, the BP neural network-based method was found to consistently have higher OP performance prediction results. The MSE of the BP neural network was 0.0018862, which is lower than the MSE of the LWLR, SVM, and ELM methods. The proposed algorithm can be used to predict the OP performance of vehicular communication systems employed in inter-vehicular communications, intelligent highway applications, and mobile ad-hoc applications.

In the future, the impact of correlated *N*-Nakagami channels on OP performance will be evaluated. The long-short term memory (LSTM) model will be considered to predict OP performance. Compared to the BP algorithm, the LSTM could offer more details on time-series and capture short- and long-term memory, adaptively reflecting environmental categories.

## Figures and Tables

**Table 1:** The parameters for the TAS scheme.

*μ*	0 dB
*K*	0.5
*γ*_th_	5 dB
*m*	1
*N*	2
*N*_t_	1,2,3
*N*_r_	2
*L*	2

**Table 2:** The parameters for the TAS scheme.

*μ*	0 dB
*γ*_th_	5 dB
*m*	1
*N*	2
*N*_t_	3,4,5
*N*_r_	1
*L*	1

**Table 3:** The parameters for the TAS scheme.

*μ*	0 dB
γ_th_	5 dB
*m*	1
*N*	2
*N*_t_	2
*N*_r_	2
*L*	2

**Table 4:** The parameters of the four different methods.

Algorithm	BP	ELM	SVM	LWLR
Parameter1	X:17	X:17	X:17	X:17
Parameter2	y:1	y:1	y:1	y:1
Parameter3	q:10	q:4750	c:1024	τ:0.30
Parameter4			g:0.0078	

**Table 5:** The running time and mean square error (MSE) comparison of the four methods.

Algorithm	BP	ELM	SVM	LWLR
RunningTime	2.92215 s	2.35641 s	365.91560 s	5.31633 s
MSE	0.0018862	0.0032144	0.0024255	0.031652
